# Embedding cultural studies in public health higher education: the role of medical anthropologists

**DOI:** 10.11604/pamj.2022.41.271.18226

**Published:** 2022-04-01

**Authors:** Mofeyisara Oluwatoyin Omobowale

**Affiliations:** 1 Institute of Child Health, College of Medicine University of Ibadan, Ibadan, Nigeria

**Keywords:** Medical anthropology, public health, socio-cultural, disease, pedagogy

## Abstract

Medical Anthropology is a body of knowledge with universal application. It bridges the gap between socio-cultural elements and public-health challenges; as a result, many medical anthropologists have raised the importance of culture in health matters. While public health pedagogy revolves around the ‘Germ Theory’ and the biomedical explanations of disease and illnesses; it is also very important to put the bio-sociocultural phenomena of health into consideration through an in-depth understanding of the social-cultural dimensions of health, healthcare and health-management. This is because ethnographic conceptions and the understanding of diseases, illnesses and wellbeing are germane to the success of public health. Embedding medical anthropological epistemology and research methods in public health higher education in Nigeria will contribute to the advancement of medical training through the use of ethnographic epistemology and methods, whereby vivid case studies of the social-cultural dimensions of public health issues would be subjected to critical discourse in the classroom. Utilizing ethnographic epistemological and methodological research cum pedagogical approaches in public health higher education will yield considerable success.

## Essay

### Introduction

Medical Anthropology as a branch of anthropology started in the 1950s and has contributed immensely to human development [1]. One of the key concerns in the field of Medical Anthropology is the culture-pattern of behaviors common to a group of humans, especially when it is related to the interconnectedness between culture and improving public health [2, 3]. Medical anthropology examines health issues in relations to the total ways of life across societies. It is usually premised on how and why people construct and deconstruct realities and lived experiences of, illness language, health, healing practices, diet, beliefs, tradition, economic practices, social organizations, rituals, values and norms, among other issues of life [4, 5]. It involves understanding human interactions with the physical and non-physical environment [6]. Medical anthropology adopts a set of shared understanding which characterizes a specific group of people, gives collective representations of their orientations and practices [7]. Asakitikpi reiterates that medical anthropology as a sub-discipline of anthropology provides the individual with a means of interpreting the world; one´s fellows and one´s life. In this orientation, culture is a set of symbols that establish basic and powerful moods and motivations that formulate a conception of the order of existence. These shared symbols constitute what a group of people agree to be “reality”. In this approach, culture provides humanity with meanings and the greatest and most definitive feature of humanness itself is the need as well as the ability for conceptualization [7]. The point stressed by Asakitikpi is that variations in human culture affect how issues of health, illnesses and diseases are perceived, practiced and managed. Thus, variations in ways of responding to health and ill-health have made Medical Anthropology an important branch of human-oriented disciplines and public health [8, 9]. The construction of the social and cultural realities of people is a key to understanding how health and health-related issues are treated. This implies that the science and art of preventing diseases, prolonging life, and promoting human health will not be complete, productive or sustainable, without considering people´s contextual understanding and experience of socio-cultural realities. The cultural understanding and interpretations of health is the core speciality of Medical Anthropology, and this makes the discipline essential to the study of public health [10, 11]. The contributions of William Caudill and Margaret Mead in 1953 to the study of Applied Anthropology in Medicine are an important milestone in the establishment of Medical Anthropology. Caudill and Mead established that the deployment of ethnography, documented cultural practices and variations and the impact of cultural differences on healthcare systems across various societies could be beneficial to public health [11, 12]. Many scholarly discourses also highlight the significance of social institutions like the family, marriage and the kinship system on the healthcare system and its environmental impact on human survival [11, 12].

Since 1966, many anthropologists have carried out researches on the area of health, illness and diseases. Although, the subfield of Medical Anthropology is a distinctive branch that is relatively new in Nigeria when compared to other branches of Anthropology, its contributions to human development are astounding. Medical Anthropology is primarily concerned with health-related issues from broader anthropological perspectives; it provides an understanding of cultural differences in health behaviors. It has also been defined as: A subfield of anthropology that draws upon social, cultural, biological, and linguistic anthropology to better understand those factors which influence health and wellbeing (broadly defined), the experience and distribution of illness, the prevention and treatment of sickness, healing processes, the social relations of therapy management, and the cultural importance and utilization of pluralistic medical systems. The discipline of medical anthropology draws upon many theoretical approaches. It is as attentive to popular health culture as bio-scientific epidemiology, and the social construction of knowledge and politics of science as scientific discovery and hypothesis testing.

It is important to note that this discourse on the importance of medical anthropology in public health education is not new, especially in the developed world, where anthropology is already a well-established discipline and adequately utilized. But in Nigeria, the discipline of anthropology is yet to be placed in its befitting position in public health (social and behavioural studies). Although, many public health schools in Nigeria teach elements of medical anthropology on the surface usually tag with the phrase “sociocultural…”, which is not sufficient. This is evident in many researchers judging “culture” related health issues from elitist perspectives and the inability of many to appropriately examine the cultural issue from both emic and ethic views that will holistically and reflectively examine health issues and proffer sustainable solutions. The holistic views would have contributed meaningfully to and sustainable and impactful public health interventions and policies. Thus, this essay stresses the importance and inclusion of medical anthropology in public health pedagogy in Nigeria.

Therefore, the inclusion of Medical Anthropology into the curriculum of public health higher education will equip trained public health practitioners with adequate skills to research and analyze public health problems holistically with the trained mind that health and management of illness are influenced by culture. Hence, medical anthropology has an important role to play to understand the context of illness and health within a culture/community for apposite intervention. It is, therefore, important to equip healthcare professionals with the theory and practice of medical anthropology, especially in Nigeria and other developing countries where culture plays an important role in health management. Additionally, embedding medical anthropology in public health education in Nigeria will also help to initiate holistic and contextual policies; thus providing apposite interventions to public health problems. The pivot of this discussion is that Medical Anthropology inclusion in the public health curriculum will embed reflexive, critical, holistic and inward-looking skills into public health training in Nigeria. It will allow for an adequate understanding of the nuances of human cultural variations and health in the public health higher education curriculum. All cultures possess both positive and negative traits that could also influence public health, positively or negatively; hence, there is a dire need for a contextual cultural understanding and the analysis of health challenges, needs and solutions in public health. The implication of this is that an in-depth and holistic grasp of culture and health is required.

### Culture and public health

Culture involves all norms and values into which an individual is socialized and acculturated, as a momentary or permanent member of a group [11, 13]. The group value system defines societies, spaces and wellbeing and its socio-cultural constructions of existence. Cultural norms and values differ across spaces and time, so also do the definitions allotted to health, illnesses and diseases. However, a constant factor that characterizes many societies is that they seek to achieve and maintain good health within the context of their cultural understanding and interpretations of health. For instance, Jegede [14] states that the view surrounding mental illness among the Yoruba speaking people of South-Western Nigeria is narrower in interpretation when compared to that of the Western culture. Mental illness in the Yoruba interpretation refers only to the manifestation of mental disorder, while mental illness in the West also deals with the primary stages of mental problems. This implies that the cultural construction of mental illness among the Yorubas of South-western Nigeria indicates a need for help or the management of the illness. In essence, culture plays a vital role in the interpretations and explanations of public health problems. Since the essence of public health is to protect and improve the health of individuals and families and communities through the promotion of healthy lifestyles, researches carried out on diseases and injury prevention, and the detection and control of infectious diseases must also include aspects of peoples´ lives and cultures which may enhance or endanger health [11, 14]. The overall goal of public health is the protection of the health of both small and large communities, an in-depth and holistic understanding of the community´s culture is an important way to achieve this goal [9, 15].

Understanding the health-related behavior of people (in terms of people´s individual and collective definitions, activities geared towards the maintenance of good health), the relationships between socio-cultural factors, and the causes of disease along with the utilization of health facilities in various societies, is germane to public health. Therefore, it is important to note that that the state of being healthy transcends the absence of illnesses and diseases, rather, it also encompasses a harmonious condition between the physical, mental and social environment. The wellbeing of an individual and society at large rests upon the prevailing cultural practices, beliefs, norms and values of the people, and the cultural standards that encapsulate their lives and livelihood [16]. Without a doubt, cultural understanding has been found to play a vital role in resolving public health problems like mental illnesses, non-communicable diseases, HIV and AIDS, among other public health issues. In addition, it has also helped in the formulation, implementation and evaluation of health policies over the years [6, 17-20]. This depicts the sub-field of Medical Anthropology as an integral part of public health and justifies the need for the adaption of relevant parts of the discipline in public health higher education pedagogy [21].

From the aforementioned, what is being emphasized is that human health is greatly influenced by daily behavioral patterns, taken for granted human actions and events, cultural and social constructions, and the interpretations and performance of realities generally referred to as culture and lifestyles. Certain behavioral patterns and practices that lead to tobacco use, physical inactivity, poor nutrition, alcohol and drug abuse, violence, unsafe sexual practices, obesity, gender discrimination, class inequality, the resistance of immunization, poor management of non-communicable diseases and risky behaviors that account for a large proportion of premature morbidity and mortality on a global scale, are all by-products of social, cultural or sub-cultural human behaviors [16, 22]. Over the last six decades, the role of Anthropology in influencing and changing human behaviors has become more prominent globally [3]. It has also helped to understand and provide explanations as to why health-related interventions will succeed in one society and fail woefully in another society, and also how health interventions can be adapted into sustainable interventions across cultures [13, 23]. Medical anthropology and other cognate disciplines have played important roles in health counseling, group-based interventions, social marketing, and policy interventions and changes [16, 24]. Hence, adapting relevant medical anthropology explicitly into public health pedagogy in Nigeria is required for the achievement of sustainable health results, that will leave no one behind. Therefore, cultural factors are central to public health issues, and the discussion so far, stresses the importance of including anthropological contents that emphasize and review the linchpin between culture, health and variations in the curriculum of public health at all levels of studies. This inclusion will enable public health professionals who would be versed in understanding the true meaning of socio-cultural issues in public health and its application, to make effective and sustainable interventions and policies that will bring about positive changes in health behaviors of local, national and global complex populations.

Those who trained to improve people´s health require an adequate understanding of social and cultural constructions of daily realities; these should be reflected in the public health curriculum through the inclusion of anthropological theories and methods. A typical example can be drawn from non-communicable diseases (NCDs), which account for 71% of premature deaths worldwide, and 82% of these occur in low-income and middle-income countries like Nigeria [25]. In Nigeria, non-communicable diseases account for 27% of deaths. Non-communicable disease in Nigeria´s content is a “socially transmitted non-communicable disease”, because many factors leading to incidence, late presentation in the hospital and poor managements of non-communicable diseases are entwined around socio-cultural issues. Issues like the poor economic status, the socialization/youth culture leading to an increasing harmful use of alcohol and drug abuse (the abuse of tramadol, codeine and alcoholic herbal mixtures) among youths are predisposing factors, which account for NCDs [26-30]. In order to combat the occurrence and prevalence of NDCs in Nigeria, and to achieve good health by 2030, there is a need to train public health professionals about the importance of identifying and relating with social and cultural practices contributing to the occurrence and prevalence of NCDs.

A good example from the recent past which further reiterates the importance of anthropology in public health pedagogy is the case of polio vaccine rejection in Northern Nigeria, where an appeal to indigenous cultural elements ensured relative success, which had been elusive, hitherto [30]. In Nigeria, the issue of childhood vaccine rejection has been a long-term problem in public health, dating as far back as 1903. Ever since the pre-colonial era, the issue of vaccine rejection has been a serious bane to the achievement of health goals. The ripple effect of the resistance entrenched Nigeria as one of the countries with the highest childhood mortality rate in the world. However, in 2015, Nigeria was declared polio-free after an appeal to indigenous cultural symbols and elements. The point being made is that courses on cultural conflicts and healthcare will be of benefit to the public health curriculum as studies on culture and health have shown that cross-cultural historical and local perceptions of health are very important not only to public health but also to human development [2] For example [17], revealed that a study on malaria and culture was carried out and showed a gender classification of malaria among the Yoruba people of South-Western Nigeria. In Yoruba land, malaria is classified as being masculine or feminine and the treatment seeking modes are tailored along these lines of classification. Ajala and Ediomo-Ubong [15] also analyzed constructions of etiology, especially the management of illnesses and diseases in connection with witchcraft´ among the Ibibios of South-South, Nigeria. The essence of recalling these studies is to foreground the relationship between culture and the role of Anthropology and its importance to the public health curriculum.

### Medical anthropology and public health pedagogy

Since 1940, medical Anthropology has helped health care providers to understand cultural differences in health behaviors. Furthermore, over the years, the discipline has developed epistemologically and methodologically. The development of Anthropology over the decades has also led to the development of theories and concepts such as “Explanatory Model Framework” (Kleinman); “Benchmark Volume”; “Clinically Applied Anthropology”; and “Critical and Reflexive Medical Anthropology” among other contributions to medical practices, which focus on the anthropology of doctor-patient interactions [12]. These contributions attest to the fact that Anthropology has a lot to contribute to public health pedagogy. The inclusion of Anthropology into public health will fill this vacuum in public health pedagogy. Public health pedagogy is built around five basic sciences, namely- epidemiology, biostatistics, environmental sciences, management sciences and behavioral sciences; on the other hand, medical anthropology is naturally grouped with other cardinal behavioral sciences, despite its contributions to public health.

The inclusion of Anthropology into public health pedagogy will also offer a more robust understanding of healthcare across societies through the inclusion of anthropological epistemology and methodological applications in public health studies. A blend of ethnography into local, and national public health pedagogy will enable trainees to possess a robust understanding of public health issues from emic and ethical perspectives. This implies that field-work or field-visit will be a part of the design that will input an all-round understanding of health and cultural variations in the study. In the same vein, the inclusion of relevant medical anthropological theories into public health pedagogy will add depth to the discipline. Theories like Phenomenology, Cultural Relativism, Symbolic Interactionism, Foucauldian Sexuality Theory, social action theory, sociation/asuwada theory and many others will broaden the understanding and interpretations of public health trainees and also reveal the differing health issues and realities of cultures. In contemporary times, the availability of community-based data is very important to public health, and the role of the anthropologist is germane to the collection of in-depth and holistic indigenous data that will through a lasting solution to health-related issues like child marriage, female genital mutilation, infanticide among others. Over the years many public health researchers have utilised anthropological methods in their studies and have reaped groundbreaking results. It has also been observed that many students in public health are in the habit of deploying “mixed methods and triangulation” while writing their final year dissertations, although, many of them lack adequate understanding of these concepts. The discussions, so far, spell out the importance of Anthropology/Medical Anthropology in public health pedagogy. I have heard positive comments from students and many others, who have infused tenets and strains of Anthropology in their research and dissertations. Some of these include; ‘...with anthropological methods, my data is now robust and more interesting than before’, ‘I now have a better insight into my research’, ‘medical anthropologists are involved in the study to make it all encompassing and enable our team to engage an in-depth community study that will lead to development’.

It is evident that Medical Anthropology is beneficial to public health and this is evident in “the ability to see culture in its proper context in the social world and how culture affects all research; the ability to pick up one minute and seemingly irrelevant detail [that usually becomes a problem solver]; independence from biomedical goals and hegemony allow medical anthropologies to add critical voices to the public health discourses and provision of objective qualitative data in an otherwise quantitative field”. Therefore, the adoption of these anthropological abilities into public health pedagogy will add an integrated and holistic approach to culture, critical perspectives and adaptations of anthropological theoretical explanations of health behaviours and qualitative methodological approaches in data collection and analysis. The deployment of medical anthropological approaches, methodology and theories in public health will bring about the appropriate and sustained formation and implementation of research and policies that are context-based, appealing, and of great impact to people across cultures. Finally, it will also allow for a sustained and timely achievement of health goals.

### Conclusion

Medical Anthropology as a sub-discipline of Anthropology possesses has been used to alleviate public health problems in many societies. Therefore, the inclusion of Medical Anthropology into public health pedagogy will enhance the realization of public health goals. The fact is that many students and teachers/professionals of public health appreciate the importance and involvement of Anthropology in Public health research. But this is not enough, a step should be taken further to include ethnographic epistemology and research methods into the Public health curriculum will complement the appreciation. Anthropology will offer a lot to public health pedagogy, if it is strategically included in the public health pedagogy, and taught by trained anthropologists. In conclusion, the inclusion of medical anthropological epistemology and methods in public health pedagogy will make studying public health holistic in the assessment of public health problems and proffer appropriate solutions that will work in line with the needs and requirements of people from different cultures, especially in multicultural nations of the world.
